# The DNA Methylome and Association of Differentially Methylated Regions with Differential Gene Expression during Heat Stress in *Brassica rapa*

**DOI:** 10.3390/ijms19051414

**Published:** 2018-05-09

**Authors:** Gaofeng Liu, Yudong Xia, Tongkun Liu, Shaojun Dai, Xilin Hou

**Affiliations:** 1 State Key Laboratory of Crop Genetics and Germplasm Enhancement, Key Laboratory of Biology and Germplasm Enhancement of Horticultural Crops in East China, Ministry of Agriculture, Nanjing Agricultural University, Nanjing 210095, China; 2014204016@njau.edu.cn (G.L.); ltk@njau.edu.cn (T.L.); 2 Total Genomics Solution (TGS) Institute, Shenzhen 518000, China; xiayudong@hotmail.com; 3 Development Center of Plant Germplasm Resources, Shanghai Normal University, Shanghai 200234, China; daishaojun@hotmail.com

**Keywords:** DNA methylation, methylome, transcription, heat stress, non-heading Chinese cabbage

## Abstract

Cytosine DNA methylation is a critical epigenetic mechanism in the silencing of transposable elements, imprinting and regulating gene expression. However, little is known about the potential role of mC in response to heat stress. To determine and explore the functions of the dynamic DNA methylome during heat stress, we characterized single-base resolution methylome maps of *Brassica rapa* and assessed the dynamic changes of mC under heat stress using whole genome bisulfite sequencing. On average, the DNA methylation levels of CG, CHG and CHH are 39.3%, 15.38% and 5.24% in non-heading Chinese cabbage (NHCC), respectively. We found that the patterns of methylation are similar to other eudicot plants, but with higher CHH methylation levels. Further comparative analysis revealed varying patterns for three sequence contexts (mCG, mCHG and mCHH) under heat stress indicating context- and position-dependent methylation regulation. DNA methylation near the TSS and TES may be closely associated with methylation-dependent transcriptional silencing. Association analysis of differential methylation and differential gene expression revealed a different set of methDEGs involved at early and late stages under heat stress. The systemic characterization of the dynamic DNA methylome during heat stress will improve our understanding of the mechanism of epigenetic regulation under heat stress.

## 1. Introduction

As sessile organisms, plants encounter a wide range of environmental stimuli and stresses. The gradual increase in ambient temperatures over the last century has affected plant growth and production globally, leading to severe losses in crop productivity [1,2,3]. Heat stress responses have been studied for decades, including signal transduction, accumulation of heat stress proteins (HSP), and transcription factors for gene expression regulation, amongst others [4,5,6]. Increasing evidence also indicates that DNA methylation and histone modification play critical roles in the regulation of gene expression in the response of plants to environmental stresses, including pathogens, drought, salinity, extreme temperatures and heavy metals [7,8,9,10,11,12,13]. Salt stress has been proven to cause DNA methylation pattern changes in rice [13,14], soybean [15], caliph medic [16] and maize [17]. A recent study also showed that cold-induced flavor loss of tomato is associated with transient changes in DNA methylation [18].

DNA methylation occurs at three different cytosine residue contexts in plants: CG, CHG and asymmetric CHH (where H = A, T or C) [19]. However, mammalian DNA methylation occurs mostly at CG sites by DNA methyltransferases (DNMTs), which catalyze the addition of methyl groups into cytosine residues and homologs. In *Arabidopsis*, CG methylation is predominantly maintained by DNA methyltransferase 1 (MET1), a conserved mammalian DNMT1 homologue [20,21,22]. High levels of CHG methylation in *Arabidopsis* are maintained by the plant-specific CHROMOMETHYLASE 3 (CMT3), whereas CHH methylation, and to some extent CHG methylation, is generally maintained by the activity of *Arabidopsis* domains rearranged Methyltransferases (DRMs) and CMT2 methyltransferase [19,23,24]. On the other hand, establishing DNA methylation is entirely dependent on DRM1/DRM2 through a complex pathway termed RNA-directed DNA methylation [25]. The content and distribution of the three methylation sites appear to vary drastically among different organisms [26,27]. Further, global DNA methylation levels amongst plant species may be potentially associated with different TE content and genome size in the various plant genomes [28,29]. Indeed, the TE-rich genome of Norway spruce (~70%) and rice (~40%), which belong to the angiosperms and gymnosperms, respectively, have much higher aggregate levels of DNA methylation than the TE-poor (~15%) *Arabidopsis* genome [29,30,31,32].

Cytosine methylation is found at transposons and other repeated sequences in a wide range of species [19,33,34]. Due to the nature of transposable elements, they are prevented from reactivation and transposition by DNA methylation [33,35]. In *Arabidopsis*, several other transposable elements were also found to be regulated by DNA methylation modification [36], and endogenous long terminal repeat (LTR)-type retrotransposons can be reactivated by DNA hypomethylation [37]. Consistent with its essential role in the maintenance of genome integrity by silencing transposons and harmful DNA, DNA methylation is also involved in the regulation of genes that are important for plant growth and development [38]. Case studies in plants have shown that the DNA methylation of promoter regions often inhibits transcription, while methylation within genes does not generally affect gene expression in the same manner [39,40]. For example, cytosine methylation of the coding parts of the AGAMOUS gene decreased the expression of itself [41]. Recent studies also showed the important role of DNA methylation in polyploid genome evolution, cold stress and seed development in *Brassica* crops [42,43,44]. Whole-genome bisulfite sequencing (WGBS) provides unbiased genome-wide DNA methylation profiles and has been successfully used in several studies to reveal elaborate patterns and functional effects of DNA methylation [26,34], including in *Arabidopsis* [40,45,46,47,48], rice [30] and tomato (*Solanum lycopersicum*) [46]. *Brassica* is a genus of 37 species of flowering plants in the mustard family (*Brassicaceae*), many of which are important agricultural crops. However, only one study has recently presented the single-base resolution of the *B*. *oleracea* methylome [49]. Further, only a limited number of studies have assessed the potential involvement of altered DNA methylation under heat stress [50,51,52].

The non-heading Chinese cabbage (*Brassica rapa* ssp. *Chinensis*, NHCC), also named bok choy or pak choi, is a subspecies of *B. rapa*, a genus containing several other morphologically-diverse subspecies including Chinese cabbage (*B. rapa* ssp. *pekinensis*), turnip (*B. rapa* ssp. *rapa*) and broccoletto (*B. rapa* ssp. *broccoletto*) [53]. *Brassica rapa* is one of six widely-cultivated *Brassica* described by the classic and well-known “triangle of U” theory [54]. Non-heading Chinese cabbage (NHCC) originated in China and is widely used as a vegetable crop due to its strong adaptability, short growth period, good quality, unique flavor and high nutritional profile. Therefore, it is widely cultivated in Southeast Asia, Japan, the USA and Europe and is becoming increasingly popular worldwide. To better understand the role of DNA methylation in *B. rapa*, we present single-base resolution DNA methylome and dynamic changes under high temperature in the non-heading Chinese cabbage cultivar “Suzhouqing” (NHCC001) using WGBS. DNA methylome and heat induced variation were analyzed. Furthermore, the methylation patterns and the potential influence on transcription were found to vary significantly across sequence contexts and genic regions. On the other side, a number of differentially-methylated regions (DRMs) and overlapping related protein-coding genes were also identified. 

## 2. Results

### 2.1. Methyltransferase Inhibitor Decreases the Heat Tolerance

**Figure 1:**
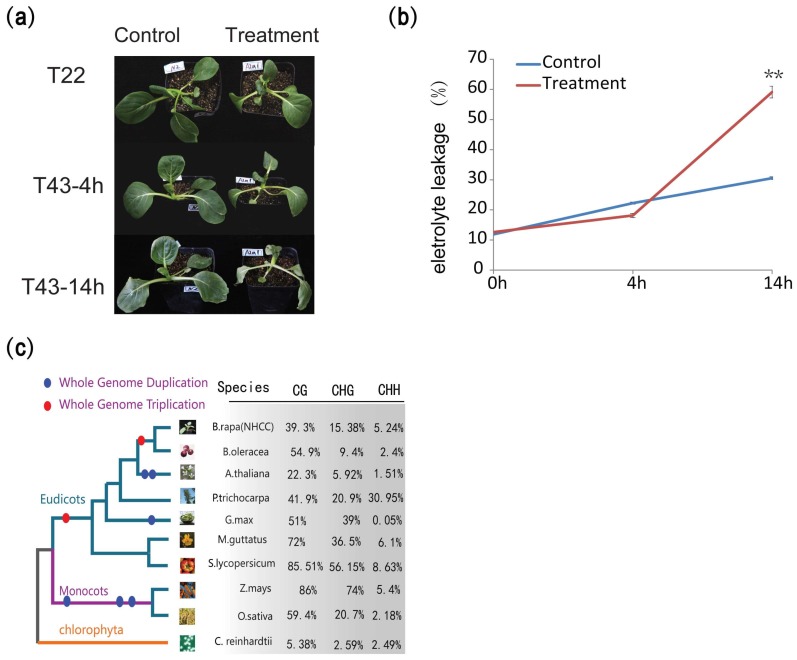
Application of a methylation inhibitor decreases the heat stress tolerance of NHCC001 and the DNA methylation landscape of non-heading Chinese cabbage. (**a**) Five-leaf stage seedlings were treated at 43 °C for 4 and 14 h. The treatment groups were pretreated with 5-azacytidine, a general inhibitor of DNA (cytosine-5) methyltransferases. Seedlings treated with 5-azacytidine (100 μmol; right, treatment) showed decreased heat tolerance, compared to the untreated seeding (left, control). (**b**) The relative electrolyte leakage was determined for the seedlings treated at 43 °C for 0, 4 and 14 h (** denotes *p*-value < 0.01, *t*-test). (**c**) Overall methylation levels in *B. rapa* and the other nine species previously studied.

To test whether DNA methylation changes in the genome influence plant heat stress resistance, we analyzed the heat-tolerance difference between plants (NHCC001) treated with 5-azacytidine and a control group. We observed that the growth of treated plants was obviously repressed (Figure 1a). In terms of relative electrolyte leakage, the electrolyte leakage was 12.660% in the control group and 12.609% in the treatment group, which indicated that the DNA methyltransferase inhibitor did not influence the electrolyte leakage of NHCC001 under normal conditions (Figure 1b). The plants pretreated with 5-azacytidine reached approximately 60% after 14 h of heat treatment, compared to about 30% for the control (Figure 1b). The plants in the treatment group became more heat sensitive than the control set after 14 h of treatment at 43 °C. The results suggest that the inhibition of DNA cytosine methylation in the genome decreased the heat tolerance of NHCC001. 

### 2.2. DNA Methylation Landscape of the NHCC Genome

To further explore the pattern of *B. rapa* DNA methylation and investigate the possibility that DNA methylation dynamically responds to high temperatures, we generated methylation maps of NHCC001 under three conditions: 22 °C (T22), 43 °C for 4 h (T43-4h) and 43 °C for 14 h (T43-14h) (Figure S2). Two independent biological replicates of the DNA methylomes for each condition were generated using WGBS, yielding an average 30× sequencing depth and nearly 123.4 million clean reads per sample, of which almost 70% could be mapped to the reference genome (Table S1). A pairwise comparison of the methylation levels at methylcytosines revealed that all six individual methylomes were well correlated (Pearson’s r = 0.9179–0.9422), as were the two replicates (Pearson’s r = 0.94; Table S2), indicating the high reproducibility of our BS-Seq results. 

**Figure 2:**
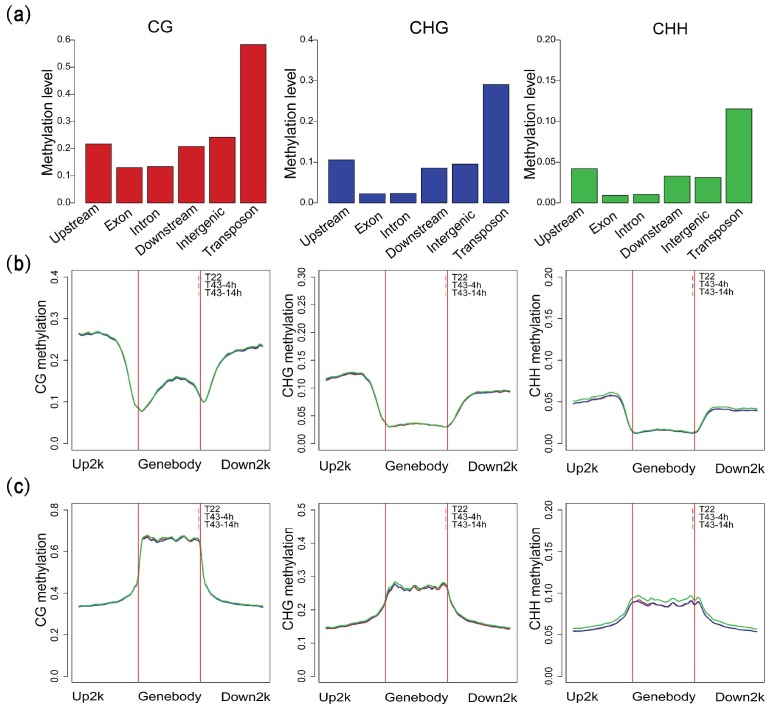
Methylation patterns of different annotated categories. (**a**) Methylation levels of different genome annotated categories; (**b**) distribution of methylation along protein-coding genes; (**c**) distribution of methylation along transposable elements (TE). Two vertical red lines mark the gene and TE boundaries. Methylation levels of upstream and downstream 2000 bp are also shown.

Under normal growth conditions (T22), 4.83% of the cytosine methylation sites were detected in 60% of the C sites (51,755,744) covered by our analysis (Table S1). We detected 758,401 mCG, 781,356 mCHG and 2,112,690 mCHH sites, and global average DNA methylation levels of CG, CHG and CHH of these mC sites were 39.3%, 15.38% and 5.24% in this study, respectively (Figure 1c). By comparing other species with reported methylation data, we found that methylation levels in NHCC were higher than those in *Arabidopsis* and *C. reinhardtii*, but lower than other species in CG context (Figure 1c). In contrast to its close relative *B. oleracea*, the levels of mCG were lower, but higher levels of mCHG and mCHH were observed in *B. rapa*. Interestingly, we found a higher methylation level of CHH than most of the other species. Recent studies also confirmed that transposable elements’ (TE) abundance and DNA methylation are positively correlated with genome sizes in plants [29,55]. Then, we also separately characterized the methylation patterns of different annotated categories in *B. rapa* by comparing the average DNA methylation level for each context. As expected, transposable elements (TE) have higher cytosine methylation levels than any other annotation element (Figure 2a), which were also consistent with transposons being a major target of DNA methylation. Protein-coding and transposon genes all harbored relatively higher levels of CG methylation than CHG or CHH methylation. Using the sliding-window approach, we found that genic regions and transposable-element regions possessed relatively unique characteristics (Figure 2b,c). The methylation levels of genic regions were similar to other flowering plants, showing a characteristic peak of the gene body of CG methylation, but not CHG and CHH methylation. Meanwhile, upstream and downstream of the gene body exhibited relatively higher methylation levels than gene-body regions. However, TE were highly methylated with CG and CHG contexts and showed an enrichment of CG and CHG methylation in the TE body compared to flanking regions. 

**Figure 3:**
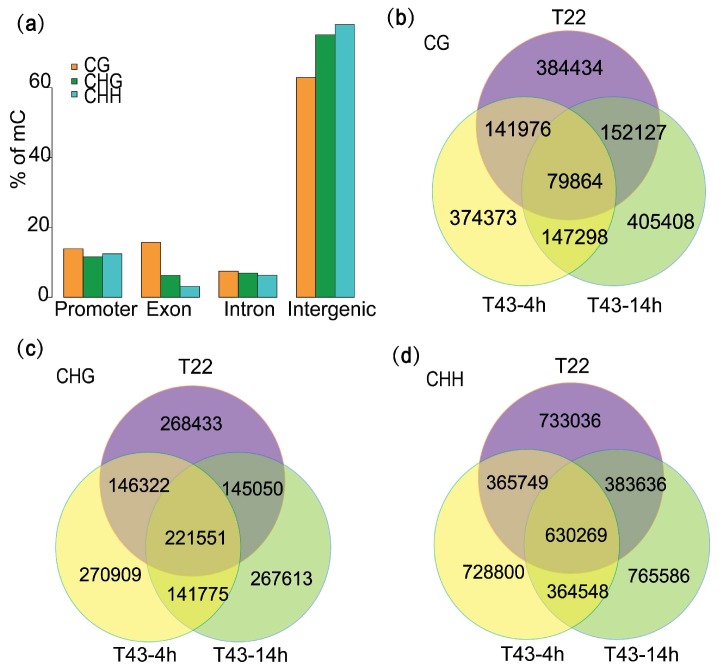
Distribution and changes of mCs under 22 °C and heat stress. (**a**) Percentage of mCs found in each genomic region; (**b**–**d**) comparison of mCs between T22, T43-4h and T43-14h for mCG, mCHG and mCHH sites, respectively.

Among all mC sites we identified, cytosine methylation occurred mostly in the asymmetric CHH sites, accounting for 57.84%, while for mCG and mCHG sites, these values were 20.76% and 21.40%, respectively. We also examined the distribution of mC sites in the genome. Among the detected methylation sites, approximately 74.3% (2,713,880) were in intergenic regions, 12.59% (459,857) were in putative promoter regions (1-kb region upstream of transcription start sites), 6.41% (234,075) were within exons and 6.7% (243,635) were in intron regions (Figure 3a). Additionally, we found a higher percentage of mCG sites (15.69%, 118,979) in exon regions, compared with the mCHG (6.28%, 49,045) and mCHH (3.13%, 66,051) sites; a converse pattern was observed in the intergenic regions. This phenomenon indicated that the distribution of symmetric and nonsymmetric methylation sites was not evenly-spread across genic and intergenic genomic sequences. 

### 2.3. Heat Stress Induces More Variation of mCG Site than the Other Two Contexts

To further examine the dynamic changes in methylation induced by high temperatures, methylation patterns were also analyzed in T43-4h and T43-14h. Overall, average CHH methylation levels alongside the protein-coding gene and TE were relatively stable under heat stress (Figure 2b,c). Upon comparing C sites between consecutive time points, we found that the quantity of mC sites detected decreased by 39,013 from T22 (3,652,437) to T43-4h (3,613,434) and increased by about 90,000 in T43-14h (3,704,725; Table S1). This trend was evident in all sequence contexts, though the quantity of mCHG sites changed to a lesser extent. The number of mCs detected in T43-4h, but not in T22 was 521,671 for mCG sites, 412,684 for mCHG sites and 1,093,348 for mCHH sites, which was slightly less than the mCs found in T22, but not in T43-4h (536,561 for mCG sites, 413,483 for mCHG sites and 1,116,672 for mCHH sites; Figure 3b–d). The number of mCs detected in T43-14h, but not in T43-4h significantly outnumbered the mCs detected in T43-4h, but not in T43-14h. These results indicated extensive demethylation events of mCs at the early heat stress stage and new methylation between T43-4h and T43-14h. We also noticed that in particular, 1,164,215 mCG sites (69.1%), 806,955 mCHG sites (55.2%) and 301,267 mCHH sites (56%) were specific to one of the three samples under different temperature conditions. Conversely, 4.7% (79,864) of mCG sites were methylated in all three samples, compared with 15.2% (221,551) and 15.8% (630,269) of mCHG and mCHH sites, respectively, observed in all conditions. Therefore, we found enormous variations in heat-induced methylation, with slightly higher rates observed at mCG sites than the other two sequence contexts.

### 2.4. Methylation Pathway Genes Are Conserved in B. rapa

To investigate the association between DNA methylation and gene expression, RNA sequencing (RNA-seq) was also performed on leaf samples of NHCC001 under three conditions: 22 °C (T22), 43 °C for 4 h (T43-4h) and 43 °C for 14 h (T43-14h), using three biological replications per condition. After 14 h of heat stress, we saw a significant heat stress phenotype and increased relative electrolyte leakage of NHCC001 (Figure 1b). The assessment of the raw data quality of nine RNA-seq libraries is shown in Table S3. After data filtering, more than 51 million clean reads were obtained from each sequence library. Then clean reads were aligned to the *B. rapa* reference genome database (http://brassicadb.org/brad/). Almost all libraries, about 80% of reads, were uniquely mapped to the reference genome. Transcripts of 37,697 genes were detected in at least one sample, with 13,219 of them significantly differentially-expressed between the three samples (Figure S1). There were more DEGs in the early stage (6843) than the late stage (4259) under heat stress, and these genes showed different change patterns during heat stress (Figure S1). Further, there were 359 and 394 genes that were constantly upregulated and downregulated during heat stress, respectively. Overall, transcriptional changes showed the rapid sense and complicated response under heat stress.

Detailed genetic studies in the model plant *Arabidopsis thaliana* have defined the key components involved in DNA methylation and demethylation pathways, such as the MET1, RNA-dependent RNA polymerase 2 (RDR2), CMT3 and DRM2 [25,56,57]. To comprehensively assess the functioning of these pathways in *B. rapa*, we searched the annotation of protein-coding genes of the *B. rapa* genome for the homologs of each of the various methylation pathway genes in *Arabidopsis* using the BlASTP method. We found that the *B. rapa* genome contains at least one copy of most key factors involved in DNA methylation control, including CMT2, CMT3, DRM2 and MET1 (Table S4). The representative genes in the RdDM pathway, such as DNA-directed RNA polymerase IV subunit 1 (NRPD1), DNA-directed RNA polymerase V subunit (NRPE1), NRPE5, RDR2, Dicer-like Endonuclease (DCL3) and Argonaute 4 (AGO4), were presented with relatively moderate expression levels in *B. rapa*. However, NPRD2, NPRD4, DML2/3 and IDL1 were missed based on the present *B. rapa* genome annotation. RNA-seq data were also retrieved to test if these genes were affected by heat stress on the transcript levels. The results showed that many of these genes involved in the RNA-directed DNA methylation pathway were heat influenced, such as upregulated HDA6, RDR2, LDL1/2, VIM1, NRPE5, SUVH2/9 and DDM1 (Table S4). Taken together, our data suggest that DNA methylation pathways are largely conserved to be functional and heat influenced in *B. rapa*, which is also consistent with the diverse changes of DNA methylation under heat stress.

### 2.5. DNA Methylation at Different Genic Regions Differentially Correlates with Gene Expression

**Figure 4:**
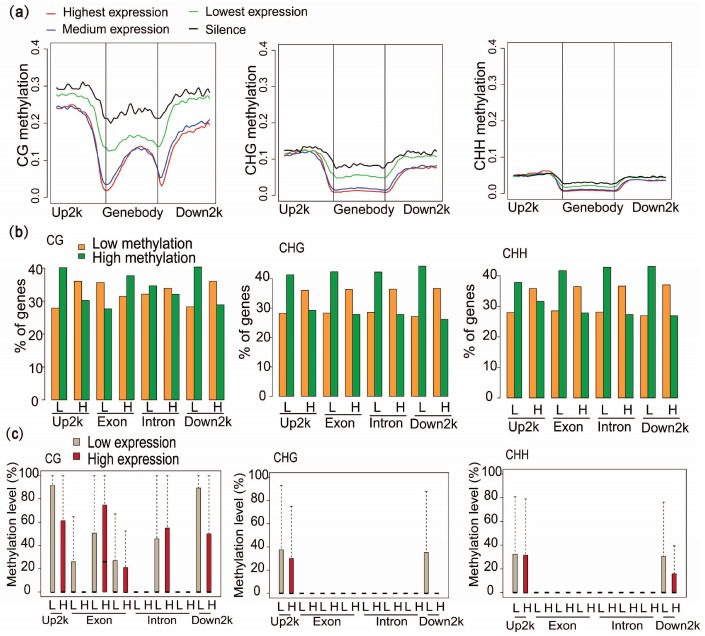
Methylation at various genic regions differentially associated with gene expression. (**a**) Association between methylation and gene expression levels in the CG, CHG and CHH contexts. Genes were sorted into four groups based on their gene expression levels. Highest expression: gene expression ≥10; medium expression: 1 < gene expression < 10; lowest expression: 0 < gene expression ≤ 1; silence: gene expression = 0. (**b**) Comparison of gene expression and methylation levels for mCG, mCHG and mCHH sites, and for each genic region: upstream 2-kb regions (Up2k), exons, introns and downstream 2-kb regions (Down2k). Low and high methylation represent the one-third of genes with the lowest and highest methylation levels with respect to each genic region, respectively. L and H at the bottom of the bars indicate the one-third of genes with the lowest or highest expression levels, respectively. Gene percentages were calculated separately for each L or H expression group. (**c**) Comparison of average methylation levels (%) between low and high expression genes for mCG, mCHG or mCHH sites in each genic region.

To assess the relationship between gene expression level and methylation level, the genes were thereafter sorted into four groups according to their expression levels. A generally negative relationship was observed between gene body methylation and gene expression levels (Figure 4a). The highest expressed genes have lower average gene-body methylation levels than the lowest expressed and silenced genes. A negative relationship was also found between promoter CG methylation and gene expression levels, but this was not seen for CHG and CHH methylation. To further look at these relationships in different genic regions, we then compared the number of low- and high-expressed genes with low or high methylation levels in the different genic regions with exons and introns classified according to their positions relative to the TSS and TES (Figure 4b). A comparison analysis found that low-expressed genes were enriched in genes with high methylation levels and vice versa, except for mCG methylation at exon regions (Figure 4b). We noticed that internal exons and introns of genes with high expression levels have relatively higher average methylation levels at CG sites compared to low expression genes, which is consistent with what has been found in other organisms (Figure 4c) [8,30,58]. The methylation levels of mCG in the first exons and mCHG in the downstream regions were significantly more negatively correlated with gene expression levels (Figure 4c). A previous study showed that DNA methylation in the region of the first exon is much more tightly linked to transcriptional silencing than methylation in the upstream promoter region [59]. Hence, mCG methylation near the TSS and TES may be closely associated with methylation-dependent transcriptional silencing. Additionally, we found that genes in low expression groups were relatively hypermethylated in the 1-kb upstream and downstream regions compared to genes in the low expression groups for CG and CHG contexts, particularly the mCHG contexts in the downstream regions (Figure 4c). Together, we proposed that the influences of methylation on gene expression levels varied with sequence contexts and genic regions.

### 2.6. Heat-Stress Induced DMRs Are Mainly Located at Gene-Related Regions and Almost Absent in the CHH Context

**Figure 5:**
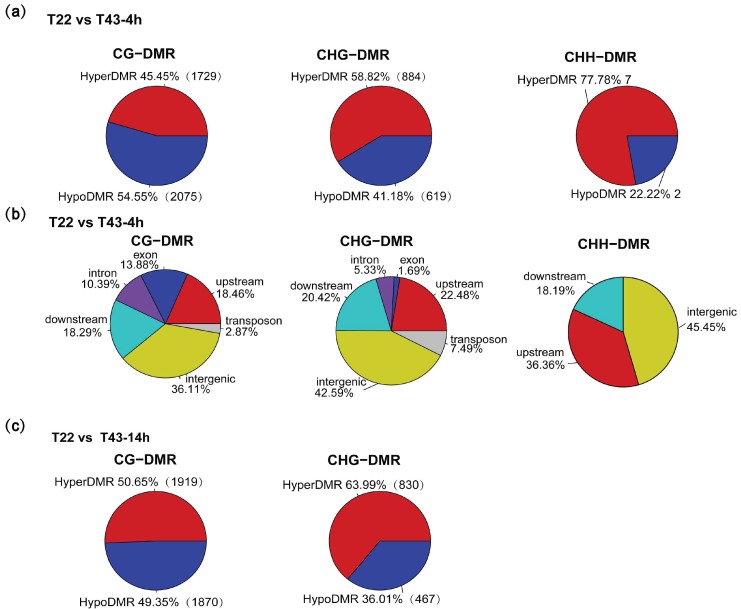

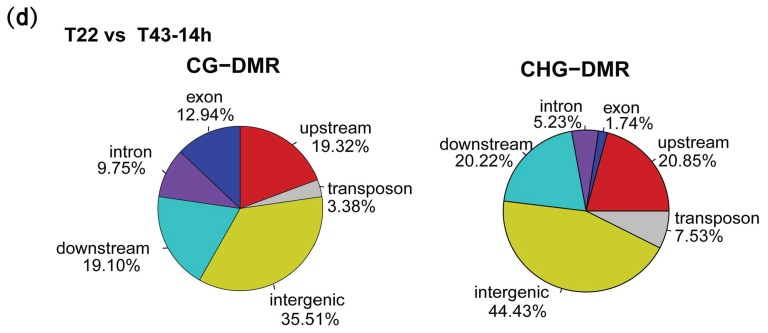
Global analysis of DMRs. (**a**) Total number of DMRs and the distribution of hyperDMRs and hypoDMRs comparing T43-4h to T22 (T22 vs. T43-4h); (**b**) CG, CHG and CHH context DMRs mapping to the different genomic categories of T22 vs. T43-4h; (**c**) total number of DMRs and distribution of hyperDMRs and hypoDMRs comparing the T43-14h to the T22 group (T22 vs. T43-14h); (**d**) CG, CHG and CHH context DMRs mapping to the different genomic categories of T22 vs. T43-14h.

In order to identify a set of heat-induced differentially-methylated regions (DMRs) under 4-h and 14-h heat stress, respectively, the regions showing significant differences in methylation levels were identified between two comparison groups: T22 vs. T43-4h and T22 vs. T43-14h (Figure 5). Under 4-h heat stress, we identified 3804 CG-related DMRs (CG-DMRs) (1729 hyperDMRs, 2075 hypoDMRs), 1503 CHG-related DMRs (CHG-DMRs) (884 hyperDMRs, 619 hypoDMRs) and 9 CHH-related DMRs (CHH-DMRs) (7 hyperDMRs, 2 hypoDMRs) (Figure 5a). Under 14-h heat stress, we identified 3789 CG-DMRs (1919 hyperDMRs, 1870 hypoDMRs) and 1297 CHG-DMRs (830 hyperDMRs, 467 hypoDMRs), and no CHH-DMRs were identified (Figure 5c). Surprisingly, only limited DMRs in the CHH context were founded under 4- and 14-h heat stress. Hyper and hypoDMR analysis also showed that hyper-CHG-DMRs were more abundant than hypoCHG-DMRs induced under heat stress. Additionally, these results revealed that high temperatures can induce hyper/hypomethylated regions, but that these vary with regards to C contexts and positions. To assess whether heat-induced DMRs are preferentially allocated in specific genomic regions, we classified these DMRs into gene-related and genomic features. We found that most of the DMRs were located at gene-related regions, also with a high-percentage proportion at intergenic regions. We also found that only small proportions of DMRs were allocated in DNA transposons. Comparison of the distribution of DMRs under different treatments demonstrated slight differences, but the overall proportions were similar: 35.51–45.45% in intergenic regions and 18.46–36.36% in the upstream (Figure 5b,d). A predominant number of gene-related DMRs induced by heat stress indicated that these dynamic changes may influence gene expression regulation under heat stress. Several randomly-selected genome regions with DMR are shown in Figure S3. 

### 2.7. Biological Processes That Are Enriched in MethDEGs Are Different between 4 h and 14 h of Heat Stress

**Figure 6:**
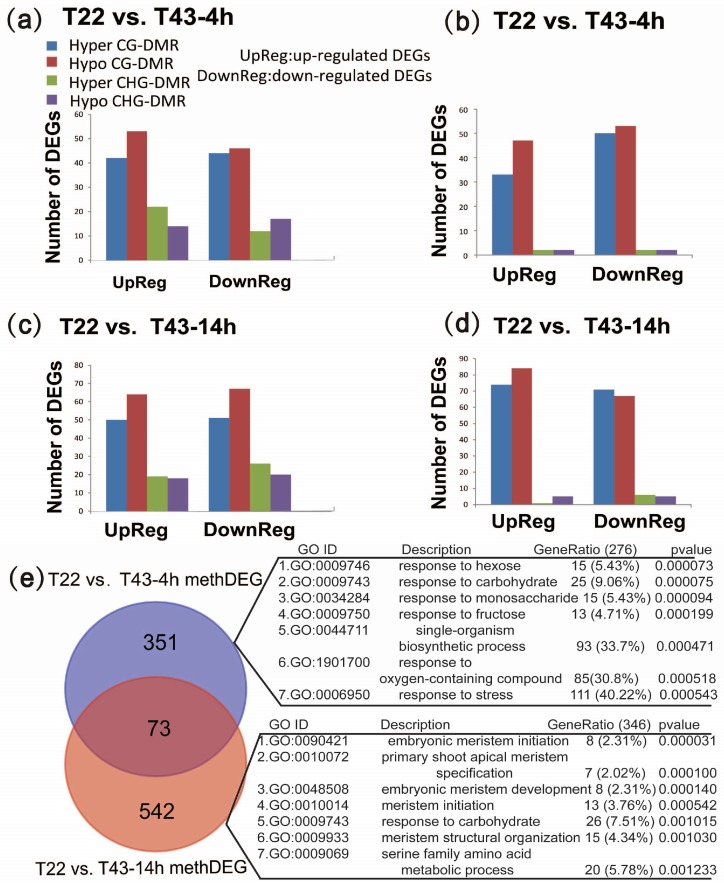
Analysis of differentially-methylated DEGs of two comparison groups (T22 vs. T43-4h and T22 vs. T43-14h). (**a**,**c**) Number of DEGs with hyper or hypoCG, CHG and CHH DMRs in promoter regions in the T22 vs. T43-4h and T22 vs. T43-14h group, respectively; (**b**,**d**) number of DEGs with hyper or hypoCG, CHG and CHH DMRs in gene bodies in the T22 vs. T43-14h and T22 vs. T43-14h group respectively; (**e**) Venn diagram of the unique and shared methDEGs between T22 vs. T43-4h and T22 vs. T43-14h. The top seven enriched GO terms (biology process) of unique gene sets are also listed.

To further investigate the potential role of the heat-induced DMRs, only DMRs that were associated with differential expression under the same heat stress condition were considered for further analysis. We found that 1561 DMRs were allocated at the gene upstream and 1397 DMRs were in the gene-body region under 4-h heat stress. Similarly, 1487 and 1345 DMRs were allocated in the gene upstream and gene-body region, respectively, under 14-h heat stress. After assigning DMRs to the gene upstream and gene body, we searched for differentially-expressed genes for which DMRs were present. Under 4-h heat stress, an association analysis showed that 243 genes with DMRs in the upstream were DEGs in our RNA-seq libraries (hereafter termed Pro-methDEGs) (Figure 6a). Ingene-body regions, 191 genes with DMRs were DEGs (hereafter termed Gb-methDEGs) (Figure 6b). Under 14-h heat stress, the number of Pro-methDEGs and Gb-methDEGs was 310 and 312, respectively (Figure 6c,d). Furthermore, we noticed that the number of hypo CG-DMRs was more abundant than other DMRs among all the upregulated methDEGs. Comparison analysis of DEGs associated with DMRs showed that at 4 h and 14 h, there were 351 and 542 unique differentially-methylated DEGs (hereafter termed methDEGs) and that a set of 73 DEGs was shared between the two sampling times (Figure 6e). GO enrichment analysis of unique and shared methDEGs between these two sets revealed that these genes were involved in significantly different biological processes (Figure 6e). Most of the methDEGs (more than 40%) were enriched in the stimulus- and stress-responding process at 4 h of heat stress, but in the development and metabolic process at 14 h of heat stress (Figure 6e). Interestingly, the set of shared methDEGs was enriched in the amino acid catabolic process. These results indicated that a different methylation program may be involved in different stages of heat stress and influence the expression of distinct gene sets to cope with heat stress over time.

### 2.8. MethDEGs Are Significantly Involved in Signaling Transduction and RNA Metabolic Process under Heat Stress 

**Figure 7:**
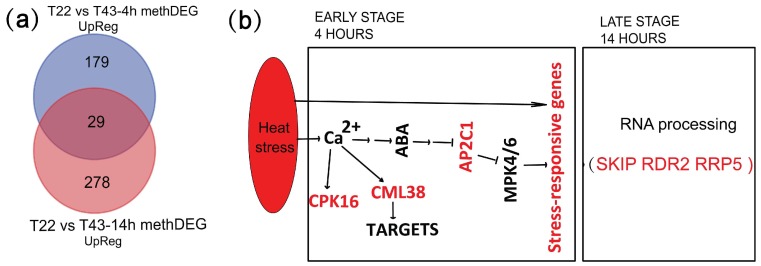
Models of methDEGs involved in heat stress response. (**a**) Venn diagram of unique and shared upregulated methDEGs between T22 vs. T43-4h and T22 vs. T43-14h; (**b**) general model of DNA methylation specifically involved in the modulation of heat stress response genes at the early and late stage of heat stress.

To get more clues about how DNA methylation was involved in the heat stress response, we examined the genes for which expression was upregulated among methDEGs. GO category enrichment analysis of the upregulated methDEGs were shown in Tables S5–S7. Among the 179 unique upregulated methDEGs, a more significant fraction of heat stress and defense-response process genes were enriched at 4-h heat stress, showing that 53 genes were involved in responding to stress (GO:0006950) and 18 were involved in temperature stimulus response (GO:0009266) (Figure 7a, Table S5). All the upregulated methDEGs are listed in Table S8. Representative methDEGs of 179 upregulated DEGs at 4 h include core components of the osmotic stress and ABA sensing signaling pathway, such as *calcium-dependent protein kinase* (*BraCPK16*, *Bra002099*) (Figure S3) and *protein phosphatease 2C* (*BraPP2C*, *Bra022772*), among others. CPK16 can be activated by Ca^2+^ signaling and further phosphorylate and activate RbohD, which generates H_2_O_2_ to induce Ca^2+^ signaling of neighboring cells by heat and other abiotic stresses [60]. The abundance of AP2C1 can regulate the activity of MPK4 and MPK6, which mediate the stress signaling and activate early-defense genes [61,62]. A set of genes, such as calmodulin-like protein (*BraCML*, *Bra015727*) (Figure S3), ubiquitin-protein ligase (*BraPUB*, *Bra023044*) and plant intracellular Ras group-related LRR 4 (*BraPIRL4*, *Bra017712*), were involved in early-defense signaling and defense processes [62,63]. Interestingly, among the 278 unique upregulated methDEGs at 14 h, genes were specifically enriched in RNA metabolism, including RNA modification and macromolecule metabolic and modification process genes at 14-h heat stress (Figure 7a, Table S6). Of particular interest is the finding of *SNW/SKI-interacting protein* (*BraSKIP*, *Bra015687*) and *RNA-dependent RNA polymerase 2* (*BraRDR2*, *Bra035249*), which were involved in alternative splicing of stress-response genes and endogenous siRNA formation, respectively (Figure S3). SKIP, as a splicing factor, was required to ensure the accurate splicing of pre-mRNAs of stress-induced genes [64]. Other genes, such as *nucleolar ribonucleoprotein protein* (*BraIMP4*, *Bra027789*) and *ribosomal RNA processing 5* (*BraRRP5*, *Bra038690*), were also involved in RNA processing. Together, these results show that methDEGs were dominantly involved in heat stress signal transduction at an early stage and in regulating RNA processing at the late stage of heat stress.

## 3. Discussion

### 3.1. Dynamic DNA Methylation under Heat Treatment

As sessile organisms, plants suffer from both abiotic and biotic stresses. Numerous studies [8,10,11,13,65,66] have reported that genome-wide changes in the DNA methylome occur in response to environmental stress. However, many of them used low-resolution and nonquantitative techniques such as methylation-sensitive amplification polymorphism, which limited the resolution to provide the specific context and genomic location of the changes in DNA methylation, thus offering limited insights into the potential role of stress-induced changes in DNA methylation. In addition, to the best of our knowledge, few studies have characterized the dynamic changes in the methylome under heat stress. Here, genome-wide characterization of the *B. rapa* methylome and dynamic changes under heat stress were carried out using WGBS, which allowed the identification of DNA methylation patterns and changes in single-base resolution. Although the general trend of the *B. rapa* methylome patterns was similar to other plant species, our analysis showed that the *B. rapa* genome has a particularly high portion of CHH methylation and that global average CHH methylation levels are much higher than *B. oleracea* and *Arabidopsis*. Global average DNA methylation level analysis in *B. rapa* also provides evidence to confirm the positive correlation between methylation levels and genome sizes.

Our analyses revealed the DNA methylation levels to be relatively stable under heat stress (Figure 2). This finding is consistent with other studies investigating the overall levels of DNA methylation under nitrogen and water-deficient conditions [67,68]. Our results also provided evidence in support of the findings of several recent studies that universal methylation-buffering mechanisms exist. We observed a dynamic trend in cytosine methylation sites characterized by an initial fall followed by a rise, with predominant demethylation of cytosine sites in the early stages of heat stress followed by new methylation. Previous studies have reported that heat stress tends to increase the overall DNA methylation levels, with heat-sensitive genotypes showing a greater increase under heat stress compared to heat-tolerant genotypes [51,69]. Previous studies have also indicated that salt and drought stress induce DNA demethylation in the leaves of tolerant cultivars [10,70]. Therefore, we hypothesized that the modulation of methylation under different stresses and conditions may be vastly different.

### 3.2. Differences in Methylation Patterns of mC Sequence Contexts and Genic Regions during Heat Stress

WGBS in single-base resolution allowed a comprehensive view of methylation patterns. In *Arabidopsis* flower buds, methylation in the CG context accounted for 55% of all methylation sites, with only 22% methylation in the CHH context [71]. However, in our study, the numbers of mCHH constituted the majority, accounting for 57.84% of all methylation sites under normal conditions. In addition, we found a higher percentage of mC in intergenic regions, which is consistent with the methylation distribution in maize roots. Conversely, in *Arabidopsis* flower tissues, a higher fraction of mC distributed in exons was observed [72]. The difference in the pattern and distribution of C methylation that exists between plant species and tissues requires further investigation. In *Arabidopsis*, natural CMT2 variation is associated with genome-wide methylation changes, and increased heat tolerance was observed in *cmt2* mutants [73]. A recent study also found that CHH methylation of TEs is temperature sensitive, and CMT2 as a major trans-acting controller of it [74]. In our study, the relatively high percentage of mCHH sites, together with heat-induced increased mCHH methylation levels along TE, suggests that CHH methylation may be important for heat stress response and tolerance. Another study also indicated that widespread dynamic CHH-methylation is important for the response to *Pseudomonas syringae* infection [8]. Thus, we can conclude that CHH is also very important for heat stress.

A detailed correlation analysis between gene expression level and methylation level allowed us to investigate the potential influence on gene expression of DNA methylation (Figure 4). Along with previous reports, the phenomenon of generally negative correlation between DNA methylation and gene expression also confirms the crucial role of DNA methylation in suppressing gene transcription. Furthermore, strong negative relationships between DNA methylation in mCG contexts in the first exon and gene expression were observed. This finding also confirmed that CG methylation in the first exon was more significantly associated with gene silencing than methylation of the nearby promoter [59]. Furthermore, we found a distant correlation between gene-body methylation in different regions and C contexts and gene expression. Together, all results suggest the existence of a more complex regulatory pattern between methylation in the gene body and gene expression.

### 3.3. DNA Methylation Is Potentially a Key Regulation Factor under Heat Stress

The abundant and detailed analysis of C methylation changes that occurred under heat stress allowed us to identify a wide range of DMRs at the whole genome-wide level. Upon comparison of two consecutive time points, we found numerous differentially-methylated regions in the genome, as well as many gene-related DMRs, which may play a potential role in the heat stress response. It is noteworthy that the intergenic regions, rather than the promoters, contained the majority of the DMRs (Figure 5). Furthermore, DMRs were almost absent in the CHH context. Considering the role of DMRs in regulating gene expression, we analyzed the DMRs located in the gene upstream and gene body. Our different time lengths of heat stress allowed us to determine a difference in DNA methylation regulation based on differing amounts of exposure time. After applying an analysis of GO enrichment to the two sets of methDEGs, we found that the differential DNA methylation regulation processes vary from the predominant involvement of stress-signal transduction to the more abundant modulation of RNA metabolism and modification, which may suggest that the fine-tuning mechanism of DNA methylation in regulating corresponding functional genes handles the influence of heat stress over time.

We concluded with a model where methDEGs may be specifically involved at early and late heat stress stages (Figure 7b). At the early stage, heat stress generates Ca^2+^ and ABA signals, which were transduced by a set of genes such as *CML38* and *AP2C1* and mediates the expression of downstream stress-responsive genes. At the late stage of heat stress, heat-induced methDEGs were predominantly involved in RNA processing. For example, *SKIP* mediates the alternative splicing of stress-induced genes and contributes to the stress tolerance of the plant. *RDR2*, which is physically associated with nuclear multisubunit RNA polymerase IV (Pol IV), is needed to generate double-stranded RNA, which is further processed into 24-nucleotide (nt) siRNA and then loaded onto ARGONAUTE (AGO4). These results shed new light on how DNA methylation is involved in the different time points of heat stress. We proposed that dynamic changes in DNA methylation play an important role in switching different sets of heat stress-induced genes in *B. rapa*. Further studies need to be done to show how DNA methylation changes are induced in specific regions and genes that are important for heat stress response and tolerance. 

## 4. Materials and Methods

### 4.1. Plant Materials and Heat Treatment Conditions

The seeds of cultivar “Suzhouqing” (NHCC001) were sown in Petri dishes that were filled with water-soaked filter paper for germination at 20 °C for 2 days. The germinated seeds of plants were planted in pots containing nutrition soil with matrix and vermiculite (3:1 ratio) directly. The plants were grown in a growth room at 22 °C, with a photon flux density of 300 μmol m^−2^ s^−1^ for 14 h/18 °C, 10 h dark cycle, with 75% relative humidity. Four weeks later, four-leaf stage seedlings under the dark cycle were moved to another growth room without light for heat treatment at 43 °C for different time lengths: 4 h and 14 h. The plants kept at normal conditions were used as control samples (CK). To detect the change of thermotolerance under methyltransferase inhibitor treatment, 200 mL of 100 μmol 5-azacytidine (Selleckchem, Houston, TX, USA, AZA#S1782) aqueous solution were sprayed on leaves beginning at 15 days after seeding (two-leaf stage), and spraying was repeated every three days in the following 10 days [75]. The control group was sprayed with water at the same time points. Materials for DNA and RNA extraction were harvested from plants without methyltransferase inhibitor treatment under CK and heat treatment conditions (43 °C for 4 h and 14 h). The mixture materials for each sample were collected from the leaves of five seedlings, which were used for DNA and RNA extraction.

### 4.2. Electrolyte Leakage Analysis

Electrolyte leakage analysis has been shown to be a reliable, quantitative and reproducible assay to predict thermotolerance in a variety of plants [76,77]. An electrolyte leakage analysis was performed using a conductivity meter (Rex DDS-307A, INESA, Shanghai, China). The samples were collected from plants under CK and heat treatment conditions (43 °C for 4 and 14 h) using a 7-mm punching bear. Half a gram of each sample was incubated at 23 °C in 10 mL glass bottles containing 6 mL deionized water and vacuumized to 0.05 MPa for 20 min. After incubation at 22 °C with gentle shaking for 3 h, electrolyte leakage (*R*1) was measured. The samples were then boiled for 20 min, and the total leakage (*R*2) of bathing solution was then measured after cooling. Electrolyte leakage (*E*) was calculated from the following equation: *E* = *R*1/*R*2 × 100. Three technical replicates were performed in each condition.

### 4.3. DNA Isolation and Whole-Genome Bisulfite Sequencing

The cultivar NHCC001 was used for WGBS and methylome analysis. Two biological replicates were used for CK and heat-treatment conditions (43 °C for 4 and 14 h). Genomic DNA was extracted from the samples using a QIAamp DNA Mini Kit (Qiagen, Germantown, MD, USA), following the manufacturer’s instructions, and quantified using an Agilent 2100 spectrophotometer(Agilent Technologies, Palo Alto, CA, USA). For BS-seq library construction, genomic DNA was fragmented by sonication to a mean size of approximately 200–300 bp using a Diagenome sonicator (Covaris, Woburn, MA, USA). Adenylation on the 3′-end and blunt-end adaptor ligation were performed according to the manufacturer’s instructions (Illumina, San Diego, CA, USA). Bisulfite conversion of the DNA was achieved using a ZYMO EZ DNA Methylation-Gold kit (ZYMO Research, Irvine, CA, USA) in conjunction with EpiMark HotStart Taq (NEB, Ipswich, MA, USA) according to the manufacturer’s protocol. Adaptor-ligated DNA was enriched by eight cycles of PCR with the following thermal profile: an initial incubation at 95 °C for 2 min, eight cycles at 95 °C for 30 s, 65 °C for 20 s, 72 °C for 45 s and a final incubation at 72 °C for 7 min. The reaction products were then purified with the Qiagen gel purification kit (Qiagen, Germantown, MD, USA) and quantified by Qubit HS dsDNA kit (Life Technologies, Carlsbad, CA, USA). Agilent 2100 was used to test the integrity of the sequencing library. After that, paired-end sequencing was performed using the ultra-high-throughput Illumina Hiseq 2500 PE 125 platform according to the manufacturer’s instructions. Two replicates per group and a 30× sequence depth for WGBS were based on a previous study [78]. Integrative Genomics Viewer (IGV) was used to visualize the DMR [79].

### 4.4. Data Filtering and Identification of Methylated Cytosines

Clean data were obtained after filtering low quality reads containing more than 50% of nucleotides with Phred quality value < 30 or N nucleotides > 10%. All clean reads were subsequently mapped to the *B. rapa* reference genome sequence (v1.2) (http://brassicadb.org) using BSMAP-2.73 software in a blasting parameter (-m 100; -x 500) [80]. After mapping, cytosine methylation sites were determined and methylation levels were calculated using BSMAP and housekeeping software. At each reference cytosine, the binomial distribution was used to identify whether at least s subsets of the genomes within the sample were methylated, using a 0.01 FDR-corrected *p*-value. Each context of methylation was considered independently: CG, CHG or CHH (where H = A, C or T). We identified methylcytosines while keeping the number of false-positive methylcytosine calls below 1% of the total number of methylcytosines that we identified. The probability *p* in the binomial distribution B (*n*, *p*) was estimated from the number of cytosine bases sequenced in reference cytosine positions in the unmethylated Lambda genome (referred to as the error rate: nonconversion plus sequencing error frequency). The selection criterion for merging two biological replicate cytosine sites was the presence of a C site in two duplicates in effective coverage (10× or more) and that the determination of methylation and unmethylation was consistent across two biological repeats. *p*-values were obtained by Fisher’s exact test, and the false discovery rate (FDR) was calculated using the Benjamini–Hochberg method [81]. Methylomes of another nine species were referenced to previously-published papers [26,46,49,82,83,84].

### 4.5. Identification of Differentially-Methylated Regions and Differentially-Methylated Genes 

Putative DMRs were identified as previously described [85]. A two-tailed Fisher’s exact test was used to identify differentially-methylated genic or promoter regions between comparison groups. The resulting *p*-values were corrected using the Benjamini–Hochberg method. Putative CG-, CHG- and CHH-DMRs were identified by comparing T22 to T44-4h and T44-14h methylomes using windows that contained at least 5 CG, CHG or CHH sites with a two-fold change in methylation level and Fisher test *p*-value < 1 × 10^20^, respectively. To further characterize the heat-induced changes in DMRs, we classified all the DMRs into five different genomic categories according to the genome annotations: transposon, intergenic, upstream (2000 bp upstream from the transcription start site), gene body (exon and intron) and downstream (2000 bp downstream from polyadenylation site). To metaplot the average methylation level of the protein-coding and transposon, we used a sliding-window approach (window size, 50 base pairs (bp); step size, 50 bp) and plotted by R software. CIRCOS software was used to illustrate the genome [86]. Consensus clustering of differentially-methylated genes and the graphic representation were performed in R software (R version 3.3.2), retrieved from http://www.R-project.org [87].

### 4.6. Total RNA Isolation and Transcriptome Analysis

Cultivars NHCC001 under CK and heat-treatment conditions (43 °C for 4 and 14 h) were also used for transcriptome analysis. Total RNA was extracted from 100 mg of plant leaf material using TRIzol reagent (Invitrogen, Carlsbad, CA, USA) according to the manufacturer’s protocol. Three biological replicates were used for CK and heat treatment. The RNA was reverse transcribed into cDNA using the Prime Script RT reagent Kit (TaKaRa, Kyoto, Japan). The cDNA libraries were constructed using an mRNA-seq assay with a fragment length range of 200 bp (±25 bp). Finally, the library was sequenced for paired-end reads of 90 bp using the Illumina HiSeq™ 2000 platform. The Illumina RNA sequencing raw datasets of CK and heat-treatment conditions (43 °C for 4 h) were extracted from a previously-published paper and re-analyzed using the following method [88]. Clean reads were mapped to the reference genome using the HISAT program [89]. Htseq-count scripts developed in Python were used to compute the number of reads per gene. Reads per kilobase of the exon model, per million mapped reads (RPKM), was used to quantify gene expression. Analysis of differential expression was performed mainly using the R packages DEGseq and DEseq [89,90]. The criteria for identifying DEGs were fold change ≥ 2 and Q-value ≤ 0.01.

## 5. Conclusions

We reported the single-base resolution methylomes of *B. rapa* genome and dynamic changes in the global DNA methylome during heat stress. We revealed a comprehensive methylome status of *B. rapa* genome and relatively stable DNA methylation levels under heat stress conditions. Our studies indicated distinct methylation patterns with regards to the three sequence contexts and genome regions. Our analyses also provided insights into the complex relationships between gene expression and methylation alteration, as well as the potentially crucial roles of methylation under heat stress. This will be an important resource for further investigation of the epigenetic pathway that regulates heat stress response.

## Supplementary Materials

Supplementary materials can be found at http://www.mdpi.com/1422-0067/19/5/1414/s1.

## Abbreviations

WGBS	Whole-genome bisulfite sequencing
NHCC	Non-heading Chinese cabbage
TSS	Transcription start site
TES	Transcription end site
DMRs	Differentially-methylated regions
DEGs	Differentially-expressed gene
methDEGs	DEGs with DMR in the promoter or gene body
