# Supplementary material for: The DNA Methylome and Association of Differentially Methylated Regions with Differential Gene Expression during Heat Stress in Brassica rapa
Source: Int J Mol Sci. 2018 May 9;19(5):1414. doi: 10.3390/ijms19051414 (PMC5983725; doi:10.3390/ijms19051414)
Supplement: Supplementary file 1 [file ijms-19-01414-s001.zip › ijms-290994-suppl.fig.docx]

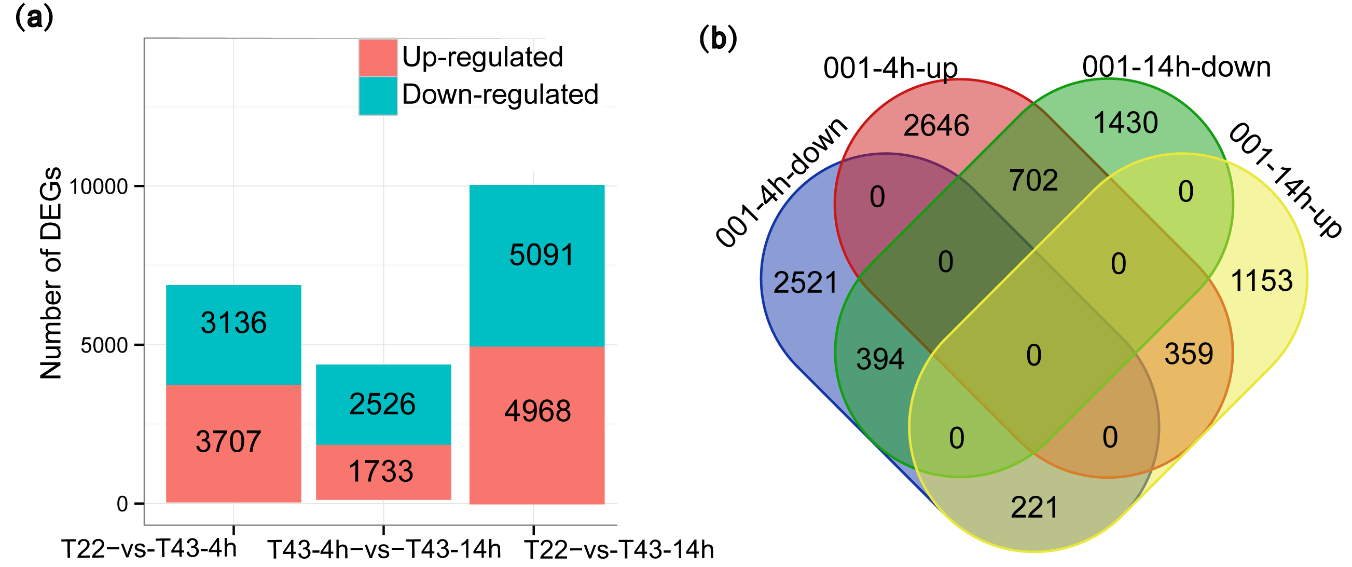


**Figure S1.** Differentially-expressed genes’ (DEG) statistics in NHCC001 under heat stress. (a) Numbers of DEGs based on the comparison of three groups. (b) Overlap of differentially-expressed genes between the four groups. 001-4h-up and 001-4h-down mean the upregulated and downregulated genes in the T22 vs. T43-4h group; 001-14h-up and 001-14h-down mean the upregulated and downregulated genes in T22 vs. T43-14h group.


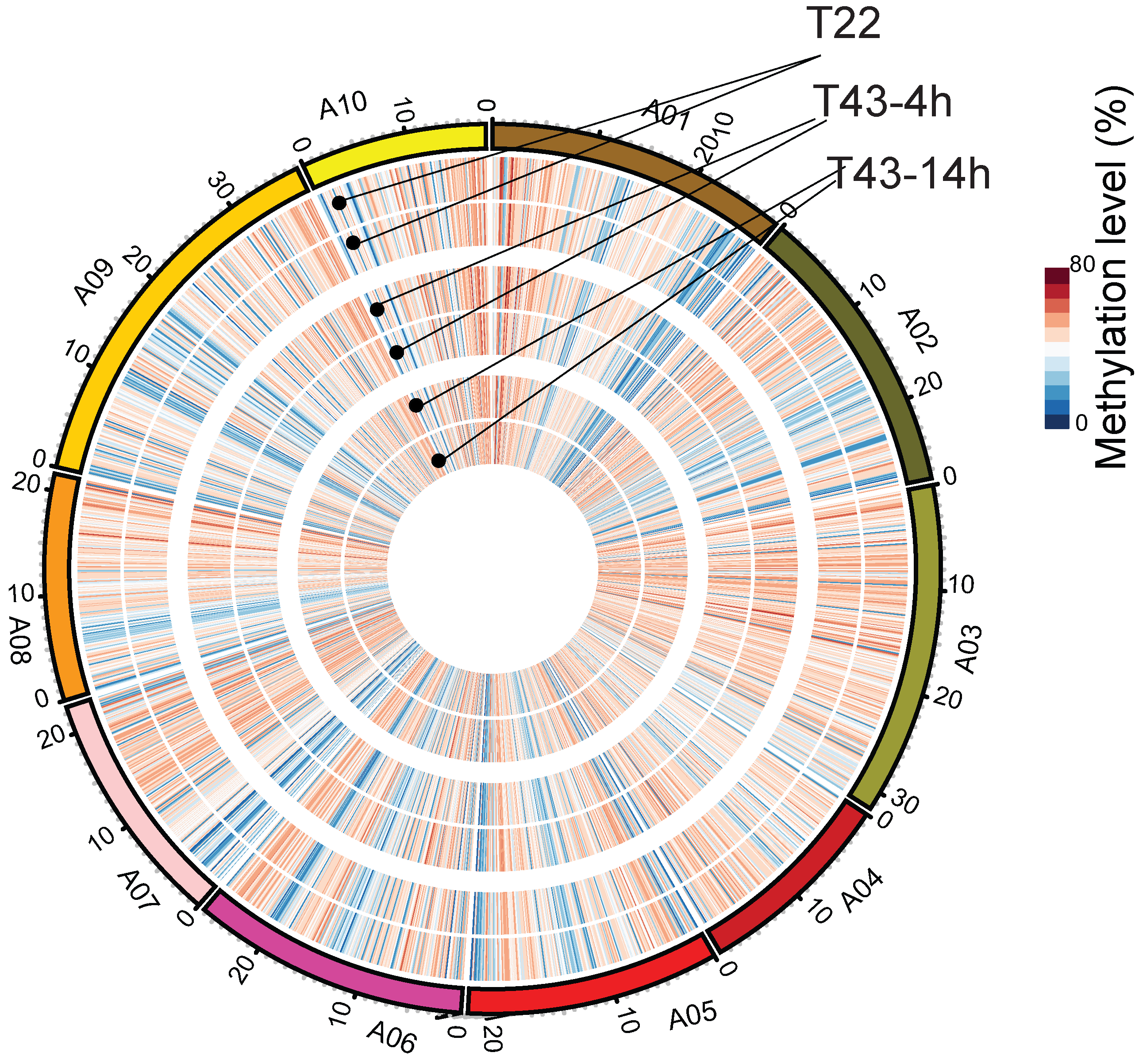


**Figure S2.** Circular representation of DNA methylation levels for leaves under different conditions. The outermost circle represents the 10 chromosomes, and the chromosome names and scales are indicated on the outer rim. The methylation levels of three conditions are represented inside the six circles. The adjacent two circles represent the two biological repeats of every condition.


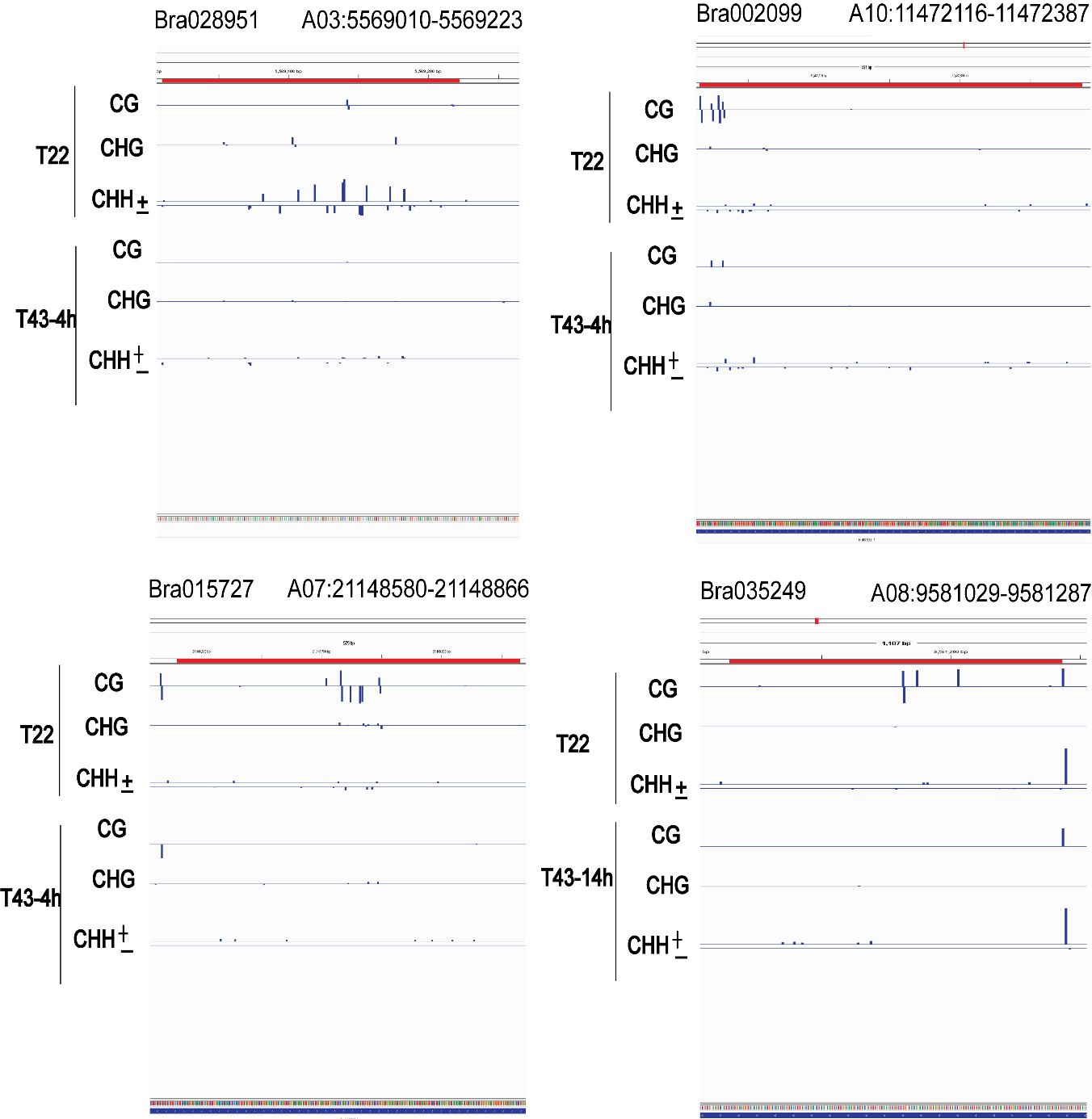


**Figure S3.** Methylation profiles of several selected gene-related DMRs using IGV. The red line on the top indicates the DMR. The blue bar indicates the methylation level.
